# Hypoxia-induced activation of HIF-1alpha/IL-1beta axis in microglia promotes glioma progression via NF-κB-mediated upregulation of heparanase expression

**DOI:** 10.1186/s13062-024-00487-w

**Published:** 2024-06-11

**Authors:** Jinchao Si, Jingya Guo, Xu Zhang, Wei Li, Shen Zhang, Shuyu Shang, Quanwu Zhang

**Affiliations:** 1 Department of Neurology, the Second Affiliated Hospital of Zhengzhou University, Zhengzhou, 450014 China; 2 Department of Neuroelectrophysiology, Zhengzhou Central Hospital Affiliated to Zhengzhou University, Zhengzhou, 450007 China; 3 Department of General Practice, the Second Affiliated Hospital of Zhengzhou University, Zhengzhou, 450014 China; 4 Department of Physiology, School of Basic Medicine, Zhengzhou University, Zhengzhou, 450066 China; 5 Department of Physiology, Medical College, HuangHe Science and Technology University, Zhengzhou, 450064 China; 6 Department of Pathology, Zhengzhou Central Hospital Affiliated to Zhengzhou University, No. 16 Tongbai North Road, Zhengzhou, Henan Province 450007 China

**Keywords:** Hypoxia, Glioma, Microglia, Hypoxia inducible factor-1α, Interleukin-1β, Heparanase

## Abstract

**Background:**

Glioma is a common tumor that occurs in the brain and spinal cord. Hypoxia is a crucial feature of the tumor microenvironment. Tumor-associated macrophages/microglia play a crucial role in the advancement of glioma. This study aims to illuminate the detailed mechanisms by which hypoxia regulates microglia and, consequently, influences the progression of glioma.

**Methods:**

The glioma cell viability and proliferation were analyzed by cell counting kit-8 assay and 5-ethynyl-2’-deoxyuridine assay. Wound healing assay and transwell assay were implemented to detect glioma cell migration and invasion, respectively. Enzyme-linked immunosorbent assay was conducted to detect protein levels in cell culture medium. The protein levels in glioma cells and tumor tissues were evaluated using western blot analysis. The histological morphology of tumor tissue was determined by hematoxylin-eosin staining. The protein expression in tumor tissues was determined using immunohistochemistry. Human glioma xenograft in nude mice was employed to test the influence of hypoxic microglia-derived interleukin-1beta (IL-1β) and heparanase (HPSE) on glioma growth in vivo.

**Results:**

Hypoxic HMC3 cells promoted proliferation, migration, and invasion abilities of U251 and U87 cells by secreting IL-1β, which was upregulated by hypoxia-induced activation of hypoxia inducible factor-1alpha (HIF-1α). Besides, IL-1β from HMC3 cells promoted glioma progression and caused activation of nuclear factor-κB (NF-κB) and upregulation of HPSE in vivo. We also confirmed that IL-1β facilitated HPSE expression in U251 and U87 cells by activating NF-κB. Hypoxic HMC3 cells-secreted IL-1β facilitated the proliferation, migration, and invasion of U251 and U87 cells via NF-κB-mediated upregulation of HPSE expression. Finally, we revealed that silencing HPSE curbed the proliferation and metastasis of glioma in mice.

**Conclusion:**

Hypoxia-induced activation of HIF-1α/IL-1β axis in microglia promoted glioma progression via NF-κB-mediated upregulation of HPSE expression.

## Introduction

Glioma is a tumor in the brain and spinal cord that originates from glial cells. Glioma accounts for about 2–3% of all adult tumors and is a common cancer in juveniles [1]. Epidemiologically, the annual incidence of glioma is 6 per 100,000 people. Glioma accounts for about 80% of malignant brain and other CNS tumors [2]. A population-based epidemiological research manifests that the incidence of glioma in adult males is 1.4 times that in adult females [3]. Gliomas can be classified into four grades, with the malignancy increasing from grade 1 to grade 4 [4]. Gliomas typically present with symptoms such as headaches, nausea, vomiting, cognitive and neurological dysfunction, but they may also have no obvious symptoms. Treatment options usually include surgical resection, radiation therapy, and chemotherapy [5, 6]. Although modern treatment methods have made significant progress, gliomas are still a very dangerous disease because they can grow and spread rapidly to other parts of the brain or spinal cord, and there is currently no definitive cure [7]. Despite receiving first-line treatment including surgery, radiation, and chemotherapy, the mean 5-year survival rate of patients with glioblastoma is still less than 10% [8]. Therefore, in-depth study of the molecular mechanisms related to glioma proliferation and metastasis is of great meaning for the advancement of novel glioma therapeutics.

In glioma, the rapid growth of tumor cells and insufficient blood supply often result in the occurrence of cell hypoxia [9, 10]. Some research has shown that hypoxia can stimulate the growth and spread of gliomas through a variety of pathways [11]. Under hypoxic conditions, deubiquitinase USP33 promotes glioblastoma growth and maintains stemness of glioma stem cells by stabilizing hypoxia inducible factor-2α [12]. Hypoxia-induced activation of galectin-8 promotes the survival and proliferative activity of glioma stem cells via the mTOR-TFEB signaling pathway [13]. Besides, hypoxia can activate hypoxia inducible factor-1alpha (HIF-1α) and HIF signaling pathways, thereby enhancing glioma cell growth, division angiogenesis and metastasis, while also reducing tumor cell apoptosis [14, 15]. Under hypoxic conditions, activated HIF-1α targets the microRNA-485-5p/SRPK1 axis to enhance the tumorigenic phenotypes of glioma cells [16]. However, the mechanism of hypoxia regulating glioma is still not fully understood and needs further study. Previous studies have shown that there is dynamic crosstalk between glioma cells and constituent parts of their microenvironment, including neurons, astrocytes, immune cells, and extracellular matrix. This kind of interaction exerts critical effect in glioma cell growth, infiltration of brain tissue, suppression of immune response, and induction of angiogenesis [17, 18]. Tumor-associated macrophages/microglia are a class of immune cells that exist in the tissues surrounding tumors [19]. Some studies have found that hypoxia stimulates the secretion vascular endothelial growth factor (VEGF) and matrix metalloproteinases by microglia cells, thus promoting the angiogenesis and invasiveness of glioma [20]. It has been documented that hypoxia can also alter the secretion pattern of microglia cells, causing them to secrete more growth factors and cytokines, thus increasing the aggressiveness and malignancy of gliomas [19, 21]. Therefore, the malignant growth and metastasis of glioma were at least partly attributed to the oncogenic activity of microglia cells in the context of hypoxia. Suppression of the cancer-promoting action of microglia cells under hypoxia may be an important strategy in the treatment of glioma.

Herein, we performed a variety of experiments to confirm the molecular mechanisms related to hypoxia regulating microglia and thus glioma cell proliferation and metastasis. Our findings contributed to further understanding of the modulation mechanism of hypoxic microglia on glioma cells and deliver a theoretical basis for the improvement of novel glioma therapeutics.

## Materials and methods

### Cell lines and culture

In this exploration, the human microglia cell line (HMC3; CL-0620) and glioma cells U251 (CL-0237) and U87 (CL-0238) were obtained from Wuhan Procell Life Technology Co., LTD (Procell, Wuhan, Hubei, China). HMC3 cells were preserved in Minimum Essential Medium (M8042; Sigma-Aldrich, St. Louis, MO, USA) supplemented with 10% fetal bovine serum (FBS; F8318; Sigma, St. Louis, MO, USA), and U251 and U87 cells were preserved in Dulbecco’s Modified Eagle’s Medium (DMEM; D0822; Sigma, St. Louis, MO, USA) with 10% FBS under 37 °C and 5% CO_2_.

### Hypoxic culture

For the hypoxia experiments, the HMC3 cells were hatched in a humidified Multi-gas incubator (HF100; Heal Force, Shanghai, China) for 24 h under hypoxia (1% O_2_, 5% CO_2_, and 94% N_2_) or normoxia (control; 5% CO_2_ in air) conditions. The culture mediums were collected and named H-CM and N-CM, respectively. The U251/U87 cells were cultured with H-CM or N-CM for indicated times, and then their malignant phenotypes, including proliferation, migration, and invasion, were evaluated.

### Cell transfection

The small interfering RNA (siRNA) against HIF-1α (si-HIF-1α) and its negative control (si-NC); siRNA against interleukin-1beta (si-IL-1β) and the control siRNA (si-NC); lentiviral vector (Lv) containing short hairpin RNA (shRNA) binding heparanase (HPSE) (shHPSE) and control (sh-NC); siRNA against HPSE (si-HPSE) and its negative control (si-NC) were obtained from Ribobio (Guangdong, Guangzhou, China). The siRNA sequences were listed in Table 1. The cells’ transfection was performed exploiting Lipofectamine 3000 (L3000015; Invitrogen, Carlsbad, CA, USA). Transfection of si-NC or si-IL-1β in HMC3 cells was carried out and then HMC3 cells were incubated under hypoxic condition for 24 h. The culture mediums were collected and named H-CM/si-NC and H-CM/si-IL-1β, respectively. To inhibit the nuclear factor-κB (NF-κB) signaling pathway, U251/U87 cells were exposed to recombinant human IL-1beta (rhIL-1β; kx20-1b; 10 ng/mL; Beijing Kexin Biotechnology Co., LTD, Beijing, China) for 24 h with or without pretreatment with BAY11-7085 (NF-κB inhibitor, 10 µM; S7352; Selleck Chemicals, Houston, TX, USA) for 60 min.

**Table 1:** siRNA sequences used in this study

Gene name	siRNA sequences (5ʹ to 3ʹ)
HIF-1α	AGUUCAACGACCAGUAGUCTT
IL-1β	GCGUGUUGAAAGAUGAUAATT
HPSE	CCUGAUGUAUUGGACAUUUTT
si-NC	AGUUUGACCUGCUCUCCAUTT

### Cell counting Kit-8 (CCK8) assay

U251/U87 cells were cultured in a 96-well plate and maintained in specific culture-medium (N-CM, H-CM, H-CM/si-NC, or H-CM/si-IL-1β) for indicated times (24, 48, and 72 h) under normoxic condition. Whereafter, CCK-8 reagent (10 µL/well; 96,992; Sigma-Aldrich, St. Louis, MO, USA) was mixed evenly with 90 µL DMEM and then added to the cells. The plate was placed back in the cell incubator, followed by incubation for 1.5 h away from light. The optical density value (at a wavelength of 450 nm) was assessed employing a multifunctional microplate reader (Model 680; Bio-Rad Laboratories, CA, USA).

### 5-ethynyl-2’-deoxyuridine (EdU) assay

U251/U87 cells were cultured in a 24-well plate and maintained in specific culture-medium (N-CM, H-CM, H-CM/si-NC, or H-CM/si-IL-1β) for 24 h under normoxic condition. Following this, an EdU kit (BCK-FC647; Sigma-Aldrich, St. Louis, MO, USA) was employed to evaluate cell proliferation. Nuclei were treated with EdU reagent and 4’-6-diamidino-2-phenylindole (DAPI; C1005; Beyotime, Shanghai, China), and the results were appraised utilizing a Laser confocal microscopy (DM2500; Leica, Wetzlar, Hesse, Germany).

### Wound-healing cell migration assay

U251/U87 cells were seeded in a 24-well plate and grown to about 90% confluency in conventional culture-medium under normoxic condition. To reduce the impact of glioma cell proliferation on wound-healing cell migration assay, U251/U87 cells were serum-starved and pre-treated with 10 µg/mL mitomycin C (a DNA synthesis inhibitor; 475,820; Sigma-Aldrich, St. Louis, MO, USA) for 1 h before scratching. After pre-treatment with mitomycin C, a 100 µL sterile tip was exploited to gently scrape the monolayer cells to make a scratch, and the results were photographed utilizing a microscope (Mateo TL; Leica, Wetzlar, Hesse, Germany) and named as 0 h. Following this, the culture medium was replaced with specific culture-medium (N-CM, H-CM, H-CM/si-NC, or H-CM/si-IL-1β) and the U251/U87 cells were cultured at 37 °C for 24 h. The views were caught and named as 24 h. The width of the scratch was assessed applying the ImageJ software (NIH, Bethesda, MD, USA). Wound healing rate (%) = (initial wound width at 0 h – wound width at 24 h) / initial wound width at 0 h × 100%.

### Transwell assay

Matrigel (Corning Inc., Corning, NY, USA) was applied to the upper surface of the transwell membrane (Corning Inc.,Corning, NY, USA) and preheated at 37 °C for 30 min. To inhibit cell proliferation, U251/U87 cells were serum-starved and pre-treated with 10 µg/mL mitomycin C for 1 h. Following this, U251/U87 cells were resuspended in specific culture-medium (N-CM, H-CM, H-CM/si-NC, or H-CM/si-IL-1β) and supplemented to the upper chamber. Whereafter, conventional DMEM medium encompassing 10% FBS was supplemented to the bottom chambers. Thereafter, the cells were maintained at 37 °C for 24 h and cells at bottom side of the membrane were subjected to paraformaldehyde (4%; P6148; Sigma-Aldrich, St. Louis, MO, USA) for 20 min and then exposed to crystal violet (0.5%; V5265; Sigma-Aldrich, St. Louis, MO, USA) for 10 min. Finally, the views were observed employing a microscope (Mateo TL; Leica, Wetzlar, Hesse, Germany). The invaded cells were counted applying the ImageJ software (NIH, Bethesda, MD, USA).

### Enzyme-linked immunosorbent assay (ELISA)

After 24 h of hypoxia or normoxia treatment, the tumor necrosis factor-alpha (TNF-α), monocyte chemotactic and activating factor (MCAF), interleukin-6 (IL-6), interleukin-1alpha (IL-1α), interferon-gamma (INF-γ), IL-1β, interleukin-8 (IL-8), and granulocyte macrophage colony stimulating factor (GM-CSF) levels in HMC3 culture medium were detected by Multiplex Human Cytokine ELISA Kit (EM10001; Anogen-Yes Biotech Laboratories Ltd., Mississauga, Ontario, Canada). Refer to the kit instructions for specific experimental procedures.

### Western blot

Proteins were drew from HMC3 cells, glioma cells, or tumor samples and detached through performing sodium dodecyl sulfate polyacrylamide gel electrophoresis. Following this, the proteins were moved to polyvinylidene difluoride membranes (IPVH00010; Merck Millipore, Billerica, MA, USA). After blocking, the membranes were treated with antibodies against HIF-1α (ab179483; Abcam, Cambridge, MA, USA), IL-1β (ab315084; Abcam, Cambridge, MA, USA), p-p65 (#3033; Cell Signaling Technology, Boston, MA, USA), p65 (#4764; Cell Signaling Technology, Boston, MA, USA), HPSE (DF12411; Affinity Biosciences, Changzhou, Jiangsu, China), and anti-β-actin (AF7018; Affinity Biosciences, Changzhou, Jiangsu, China) at 4 °C overnight. Whereafter, the membranes were exposed to specific secondary antibodies (S0001; Affinity Biosciences, Changzhou, Jiangsu, China) for 1 h. The protein blots were appraised employing an ECL-Plus reagent (Beyotime) and the views was evaluated using Image J software (NIH, Bethesda, MD, USA).

### Animal test

Four-week-old BALB/c male nude mice (weight 14–17 g) were acquired from Beijing Huafukang Biotechnology Co., LTD (Beijing, China). Following this, U251 cells were injected subcutaneously into the right flank of 15 nude mice. After 10 days, mice with tumors (100 ~ 150 mm^3^) were randomized into three groups (5 mice/group). The mice were treated with intra-tumorally injected of N-CM, H-CM/si-NC, and H-CM/si-IL-1β (50 µL) each week for five weeks. For the other 10 nude mice, U251 cells stably infected with Lv-sh-HPSE or Lv-sh-NC were injected into the right flank of nude mice (5 mice/group). Tumor volume was calculated every week using the formula: V (mm^3^) = (length × width^2^)/2. After 35 days of growth, animals were euthanized and the transplanted tumors were surgically removed for follow-up research.

### Hematoxylin-eosin (HE) staining

The tumor samples were subjected to paraformaldehyde (4%, P6148; Sigma-Aldrich, St. Louis, MO, USA) for 48 h. Whereafter, the samples were embedded in paraffin (76,242; Sigma-Aldrich, St. Louis, MO, USA) and cut into 5 μm thick slices. Before HE staining, tumor sections were baked at 70 °C for 30 min, and then dewaxed using xylene and dehydrated using gradient alcohol. The slices were stained with hematoxylin solution (C0105S, Beyotime, Shanghai, China) for 5 min, followed by rinse with distilled water. Following this, tumor sections were incubated with eosin solution (C0105S, Beyotime, Shanghai, China) for 1 min, followed by dehydration in gradient alcohol and transparentizing in xylene. The images were photographed using a light microscope (DM2500; Leica, Wetzlar, Hesse, Germany).

### Immunohistochemistry (IHC)

Detection of proliferating cell nuclear antigen (PCNA), E-cadherin, and vimentin was implemented on 5 µm thick paraffin slices. Tumor sections were treated with 3% H_2_O_2_ for 20 min to reduce endogenous peroxidase activity, treated with EDTA for 2 min for antigen repair, and blocked with QuickBlock™ Blocking Buffer (Beyotime) for 5 min. The slices were subjected to antibodies against PCNA (AF0239; Affinity Biosciences, Changzhou, Jiangsu, China), E-cadherin (AF0131; Affinity Biosciences, Changzhou, Jiangsu, China), and vimentin (AF7013; Affinity Biosciences, Changzhou, Jiangsu, China) at 4°C overnight. After rinsed three times with PBS, the slices were treated with horseradish-peroxidase-conjugated secondary antibodies (#S0001; Affinity Biosciences, Changzhou, Jiangsu, China) at 37°C for 45 min. Whereafter, the slices were subjected to 3,3’-diaminobenzidine tetrahydrochloride (DAB; Boster Biological Technology, Wuhan, China) staining away from light at 37 °C for 5 min. After the DAB dyeing solution was removed, slices were washed with distilled water to terminate the staining reaction and then counterstained with hematoxylin (C0105S, Beyotime, Shanghai, China). The pictures were photographed through a microscope (DM2500; Leica, Wetzlar, Hesse, Germany).

### IHC staining score

The IHC staining score was evaluated according to the percentage of positive cells and staining intensity, which were quantified by the ImageJ software (NIH, Bethesda, MD, USA). The staining intensity was scored as follows: 0 (negative), 1 (weak), 2 (moderate) and 3 (strong). The extent of staining was classified as follows: 0 (0%), 1 (1–25%), 2 (26–50%), 3 (51–75%) and 4 (76–100%) based on the percentage of positively staining cells. The IHC staining score was generated by multiplying the score of degree and intensity. The IHC staining scoring was carried out by two researchers who were blinded to the experimental grouping.

### Statistical assay

Data are displayed as mean ± standard deviation. The normality of the data was confirmed by the Shapiro–Wilk test. The F test (two groups) or Brown–Forsythe test (multiple groups) was applied to assess the equality of variances. Group comparison was performed utilizing Student’s *t*-test (two groups) or One-way Analysis of Variance (ANOVA) with Tukey’s post-hoc test (multiple groups). Statistical analysis was accomplished exploiting GraphPad Prism 8 (GraphPad Software, San Diego, CA, USA). A value of *P* < 0.05 indicates significant differences.

## Results

### Hypoxic microglia stimulates glioma cell proliferation, migration, and invasion

**Fig. 1:**
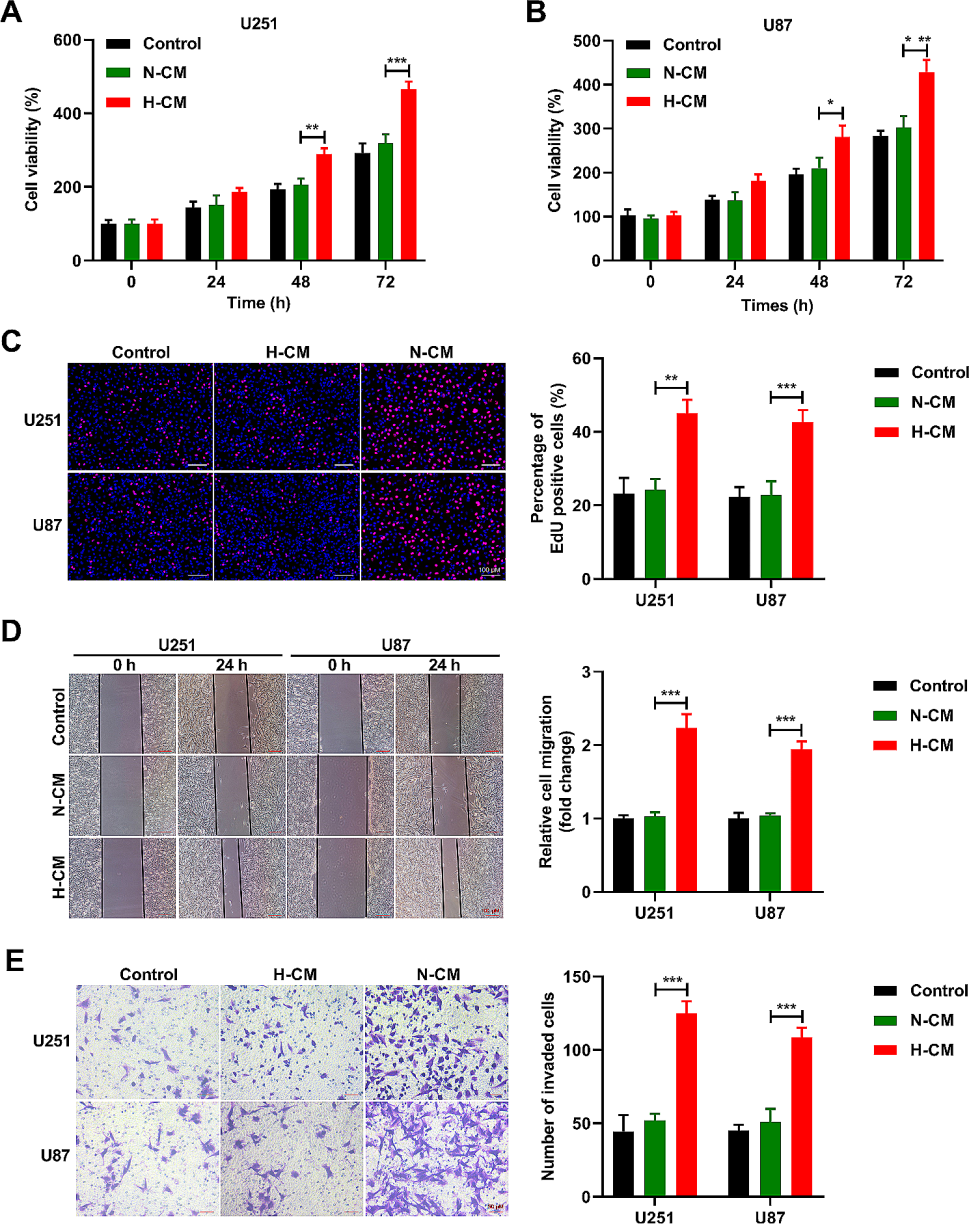
Hypoxic microglia promotes glioma cell proliferation, migration, and invasion. (**A** and **B**) The viability of U251 and U87 cells was analyzed by CCK8 assay. (**C**) The U251 and U87 cells proliferation was scrutinized by EdU assay. (**D**) Wound healing assay was implemented to detect the migration ability of U251 and U87 cells. (**E**) Transwell assay was carried out to evaluate the invasion ability U251 and U87 cells. *n* = 3. **P < 0.01, ***P < 0.001

We applied a microglia cell line HMC3 to mimic the effect of the hypoxic microenvironment on the malignant phenotype of glioma cells. Incubation of U251 and U87 cells was performed with different culture-medium for 24, 48, and 72 h. After 72 h of culture, we found that the cell viability in H-CM group was enhanced versus the control and N-CM groups (Fig. 1A and B). Moreover, we confirmed that the proliferation (Fig. 1C), migration (Fig. 1D), and invasion (Fig. 1E) abilities of U251 and U87 cells were heightened by H-CM management versus the control and N-CM groups. These results suggested that hypoxic HMC3 promoted the proliferation, migration, and invasion abilities of U251 and U87 cells.

### Hypoxia treatment stimulates IL-1β secretion by microglia through activating HIF-1α

**Fig. 2:**
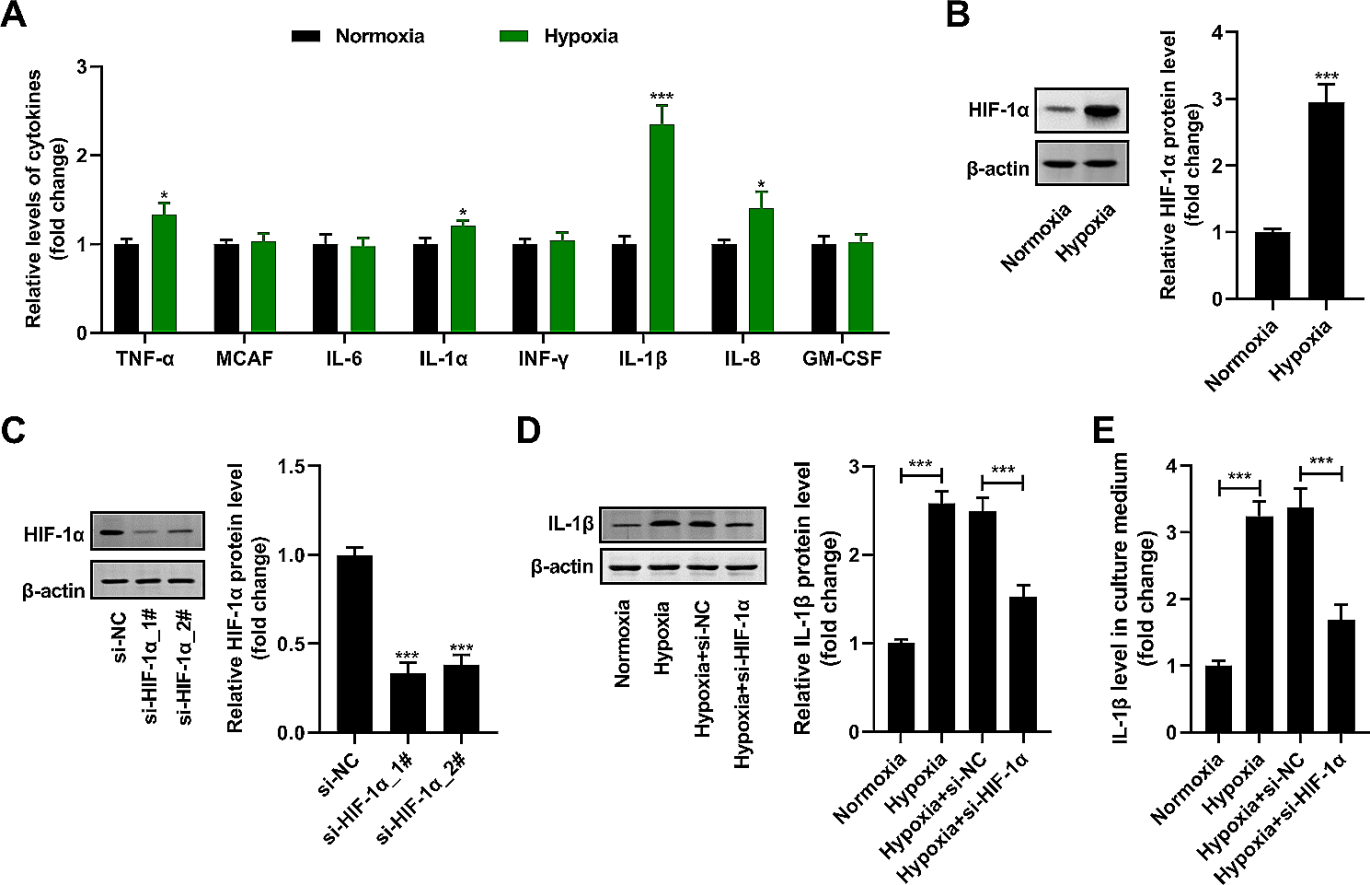
Hypoxia treatment stimulates IL-1β secretion by microglia via activating HIF-1α. (**A**) The TNF-α, MCAF, IL-6, IL-1α, INF-γ, IL-1β, IL-8, and GM-CSF levels in HMC3 culture medium were measured by an ELISA kit. (**B** and **C**) The abundance of HIF-1α protein in HMC3 cells was detected by western blot. (**D**) The abundance of IL-1β in HMC3 cells was detected by western blot. (**E**) The IL-1β level in HMC3 culture medium was detected by an ELISA kit. *n* = 3. *P < 0.05, ***P < 0.001

Herein, we revealed that the contents of TNF-α, IL-1α, IL-1β, and IL-8 in HMC3 culture medium were apparently augmented by hypoxia treatment (Fig. 2A). Among them, the rise of IL-1β is the most significant. HIF-1α protein expression was activated by hypoxia treatment in HMC3 cells (Fig. 2B). At 24 h after transfection with si-HIF-1α, the abundance of HIF-1α protein in both normoxic and hypoxic HMC3 cells decreased significantly (Fig. 2C). Moreover, the abundance of IL-1β protein in HMC3 cells (Fig. 2D) and the abundance of IL-1β in HMC3 culture solution (Fig. 2E) were boosted after hypoxia treatment, while these effects were reduced by si-HIF-1α co-treatment. Taken together, these outcomes revealed that hypoxia management heightened IL-1β secretion by HMC3 cells by activating HIF-1α.

### IL-1β from hypoxic microglia promotes glioma cell proliferation, migration, and invasion

**Fig. 3:**
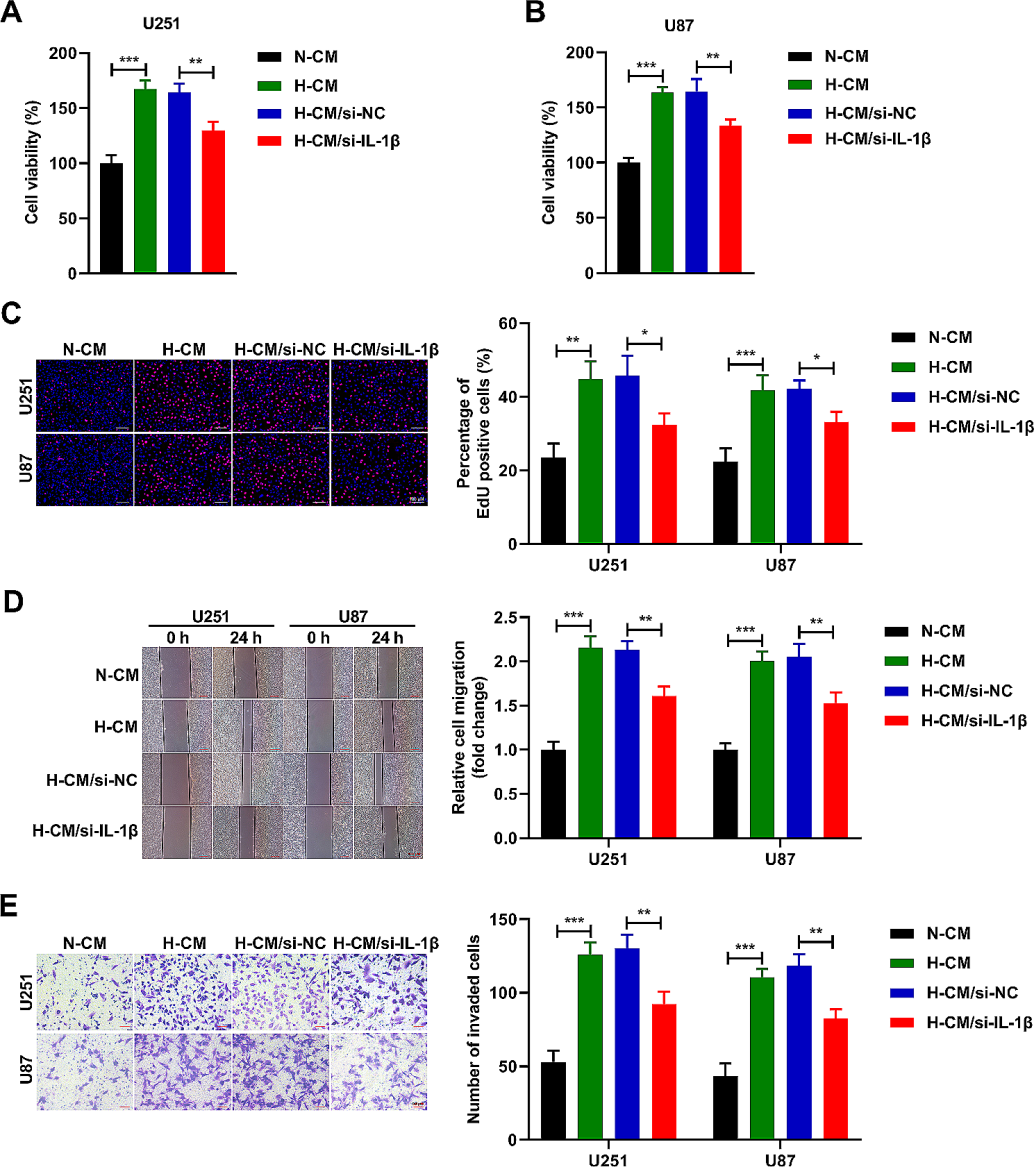
IL-1β from hypoxic microglia promotes glioma cell proliferation, migration, and invasion. (**A** and **B**) The viability of U251 and U87 cells was evaluated by CCK8 assay. (**C**) The proliferation ability of U251 and U87 cells was analyzed by EdU assay. (D) Wound healing assay was performed to evaluate migration ability of U251 and U87 cells. (**E**) Transwell assay was applied to evaluate invasion ability of U251 and U87 cells. *n* = 3. *P < 0.05, **P < 0.01, ***P < 0.001

In this part, the U251 and U87 cells were treated with N-CM, H-CM, H-CM/si-NC, and H-CM/si-IL-1β, respectively. After 72 h of culture, we detected the cell viability and found that the cell viability of U251 (Fig. 3A) and U87 (Fig. 3B) cells were boosted in H-CM and H-CM/si-NC groups, while abridged in H-CM/si-IL-1β group. After 24 h of culture, we confirmed that the proliferation (Fig. 3C), migration (Fig. 3D), and invasion (Fig. 3E) abilities of U251 and U87 cells were heightened by H-CM management versus the N-CM group, while these effects were lessened by si-IL-1β co-treatment. These conclusions proved that IL-1β from hypoxic HMC3 cells promoted the proliferation, migration, and invasion abilities of U251 and U87 cells.

### IL-1β from hypoxic microglia promotes glioma progression and causes activation of NF-κB and upregulation HPSE in vivo

**Fig. 4:**
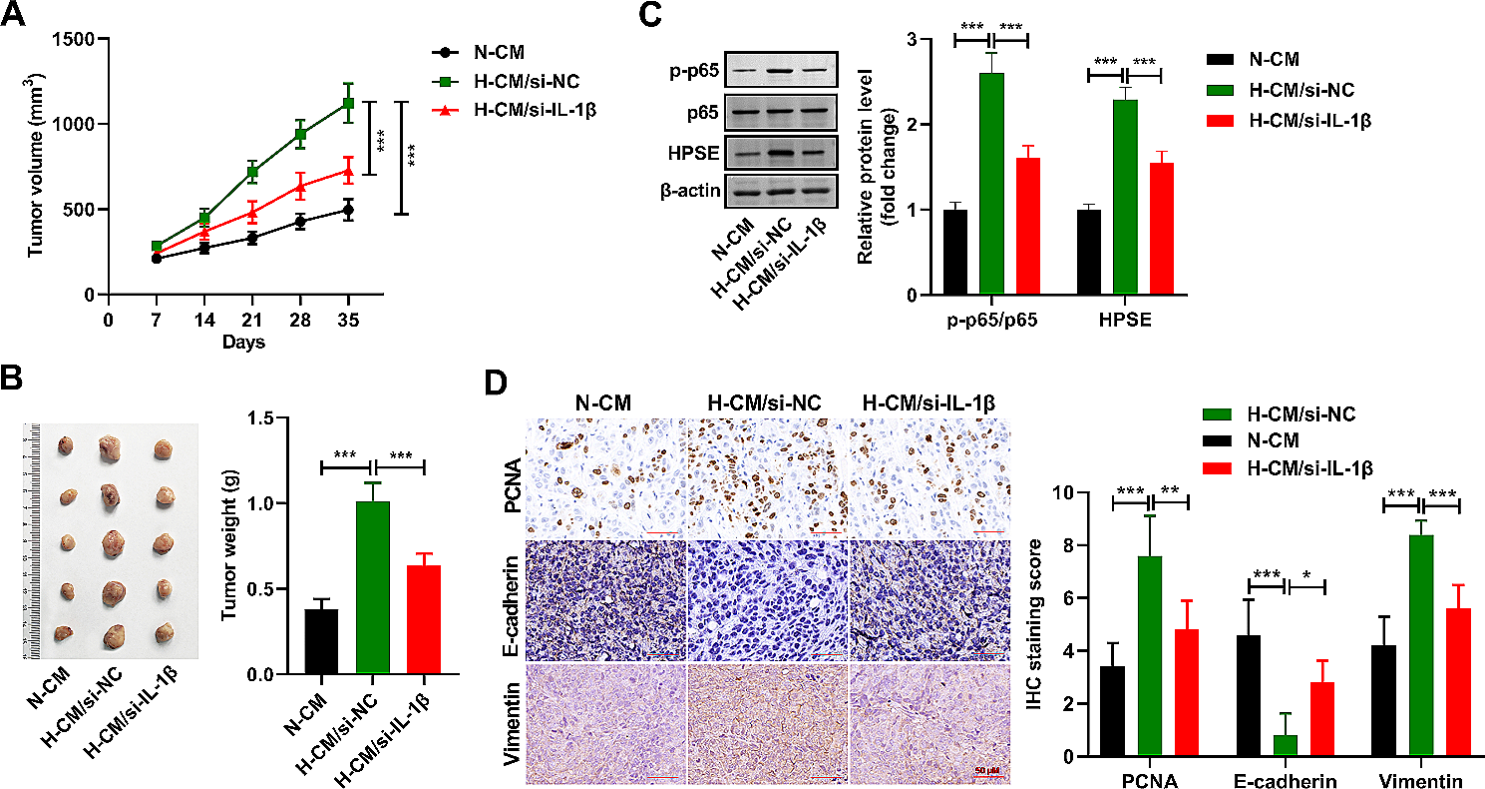
IL-1β from hypoxic microglia promotes glioma progression and causes activation of NF-κB and upregulation of HPSE *in vivo.* (**A** and **B**) Changes in tumor volume and weight were shown. (**C**) Relative protein levels of p-p65, p65, and HPSE, as quantified using western blot. (**D**) The histological morphology of tumor tissue was detected by HE staining. Relative protein levels of PCNA, E-cadherin, and vimentin, as quantified using IHC. *n* = 5. *P < 0.05, **P < 0.01,***P < 0.001

In this part, we built a xenograft tumor mouse model by injecting U251 cells, and the mice received N-CM, H-CM/si-NC, and H-CM/si-IL-1β treatment for five weeks. The tumor volume (Fig. 4A) and weight (Fig. 4B) were enhanced in the H-CM/si-NC group versus the N-CM group, while it was abridged in the H-CM/si-IL-1β group. In addition, the contents of p-p65 and HPSE were heightened in the H-CM/si-NC group in comparison with the N-CM group, while they were declined in the H-CM/si-IL-1β group (Fig. 4C). IHC results displayed that H-CM/si-NC treatment caused an increase in the PCNA and vimentin expression and a decrease in the E-cadherin expression in tumor cells. Moreover, in contrast to the H-CM/si-NC group, PCNA and vimentin protein levels in tumor cells were decreased, but E-cadherin protein level was increased in the H-CM/si-IL-1β group (Fig. 4D). These data confirmed that IL-1β released from hypoxic microglia promotes the progression of glioma and leads to the activation of NF-κB and upregulation of HPSE in vivo.

### IL-1β increases HPSE expression in glioma cells by activating NF-κB

**Fig. 5:**
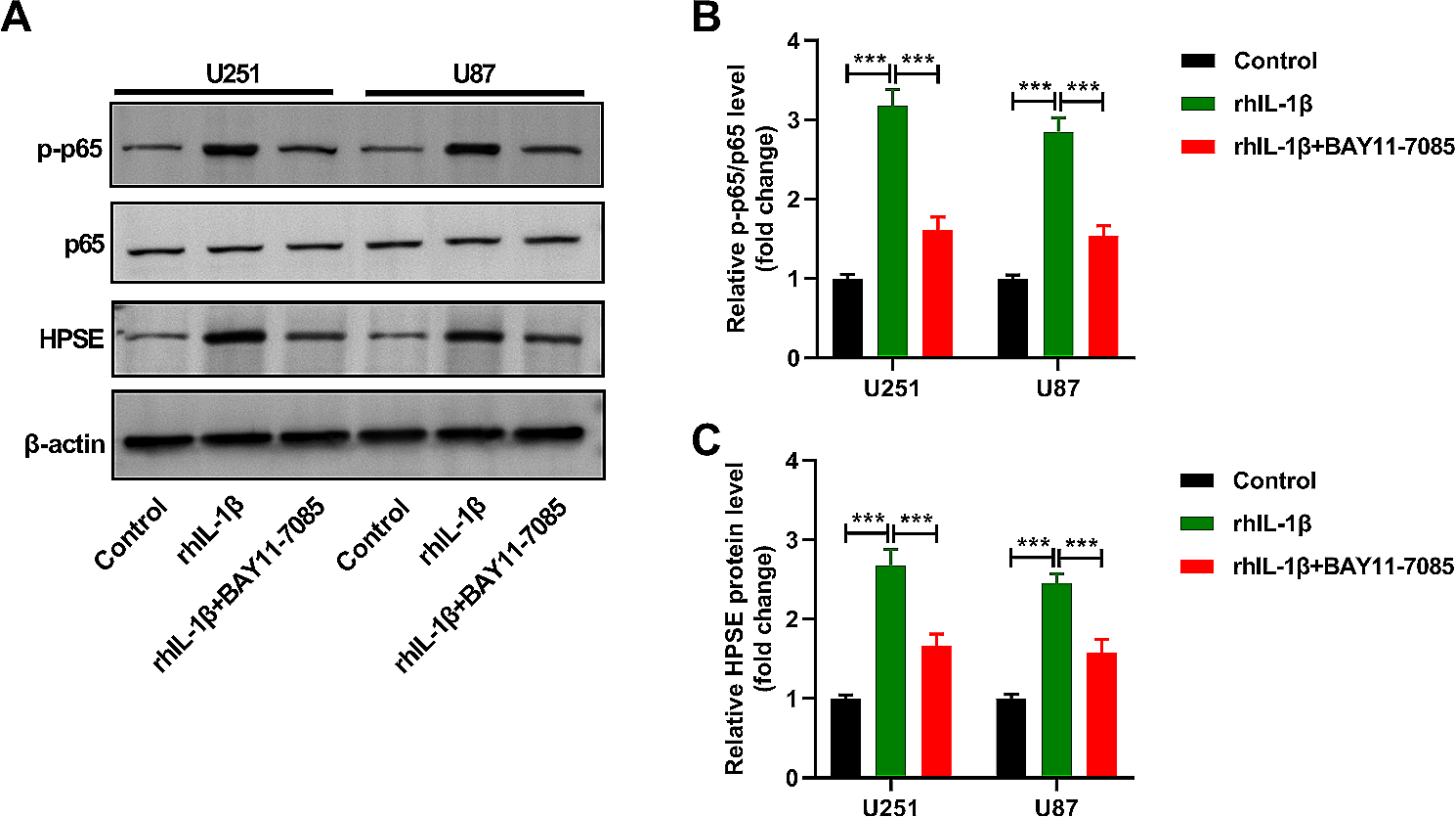
IL-1β increases heparanase expression in glioma cells by activating NF-κB. (**A** and **B**) The level of p-p65/p65 in U251 and U87 cells was monitored by western blot. (**A** and **C**) The abundance of HPSE protein in U251 and U87 cells was evaluated by western blot. *n* = 3. ***P < 0.001

Herein, we further verified the moderating relationship between IL-1β from HMC3 cells and NF-κB in glioma cells. To constrain the NF-κB signaling pathway, U251/U87 cells were treated with rhIL-1β for 24 h with or without pretreatment with BAY11-7085 for 60 min. We found that the levels of p-p65/p65 (Fig. 5A, B) and HPSE (Fig. 5A, C) in U251 and U87 cells were enhanced after rhIL-1β treatment, but these effects were lessened by BAY11-7085 co-treatment. Therefore, we revealed that IL-1β facilitated HPSE expression in U251 and U87 cells by activating NF-κB.

### Hypoxic microglia-secreted IL-1β contributes to glioma cell proliferation, migration, and invasion via NF-κB-mediated upregulation of HPSE expression

**Fig. 6:**
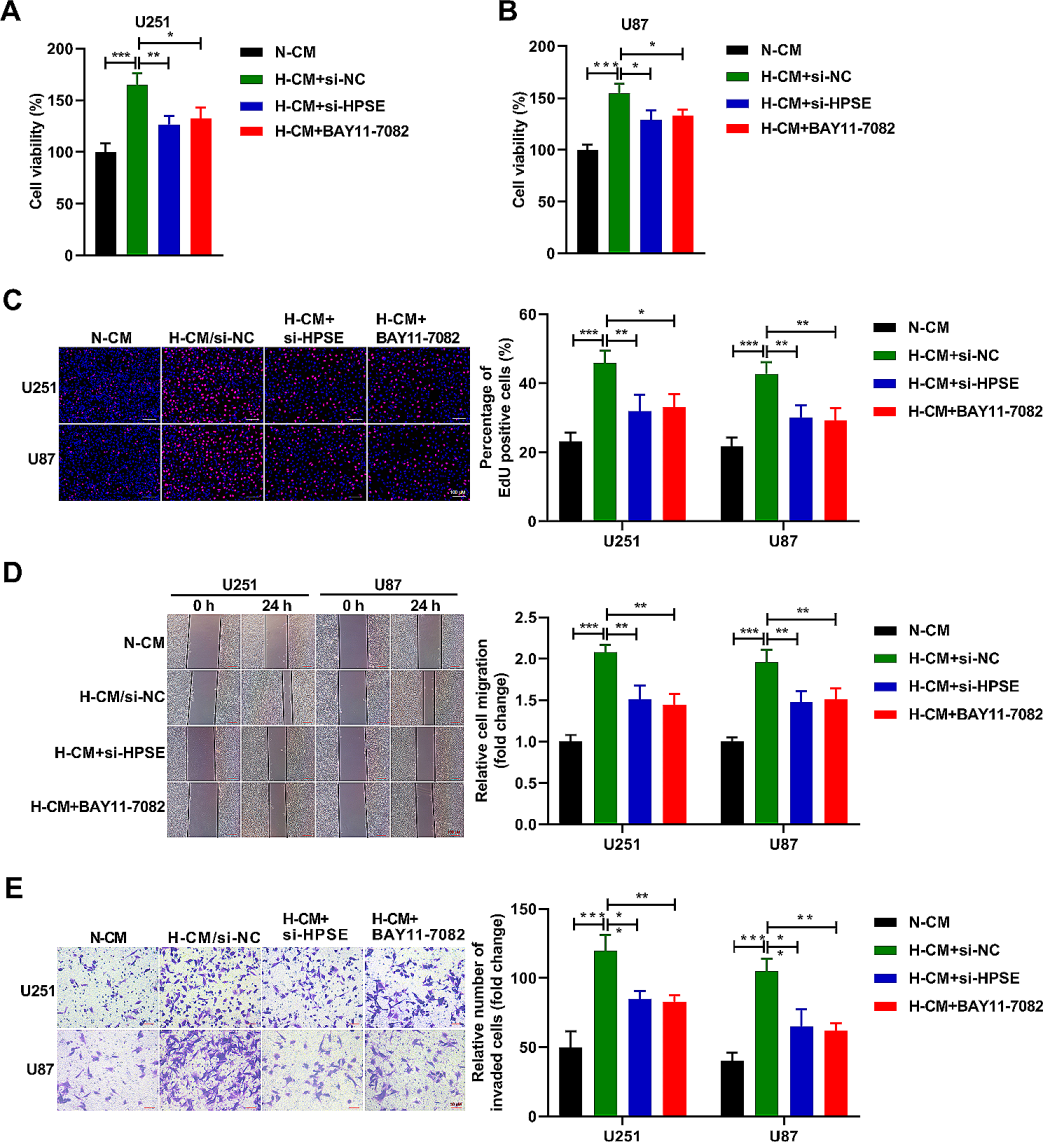
Hypoxic microglia-secreted IL-1β contributes to glioma cell proliferation, migration, and invasion via NF-κB-mediated upregulation of HPSE expression. (**A** and **B**) The viability of U251 and U87 cells was estimated by CCK8 assay. (**C**) The proliferation ability of U251 and U87 cells was investigated by EdU assay. (**D**) Wound healing assay was implemented to appraise the migration ability of U251 and U87 cells. (**E**) Transwell assay was accomplished to appraise the invasion ability of U251 and U87 cells. *n* = 3. *P < 0.05, **P < 0.01, ***P < 0.001

In this part, U251/U87 cells were transfected with si-NC or si-HPSE in the presence or absence of pretreatment with BAY11-7085 for 60 min (10 µM). Thereafter, U251 and U87 cells were cultured with N-CM or H-CM for 72 (CCK8 assay) or 24 h (Edu, wound healing, and transwell assay). We confirmed that the viability (Fig. 6A, B), proliferation (Fig. 6C), migration (Fig. 6D), and invasion (Fig. 6E) abilities of U251 and U87 cells were heightened in the H-CM + si-NC group in contrast to the N-CM group, while these effects were lessened by si-HPSE or BAY11-7085 co-treatment. These consequences proved that hypoxic HMC3 cells-secreted IL-1β facilitated the proliferation, migration, and invasion abilities of U251 and U87 cells via NF-κB-mediated upregulation of HPSE expression.

### Knockdown of HPSE inhibits tumor growth of glioma cells in vivo

**Fig. 7:**
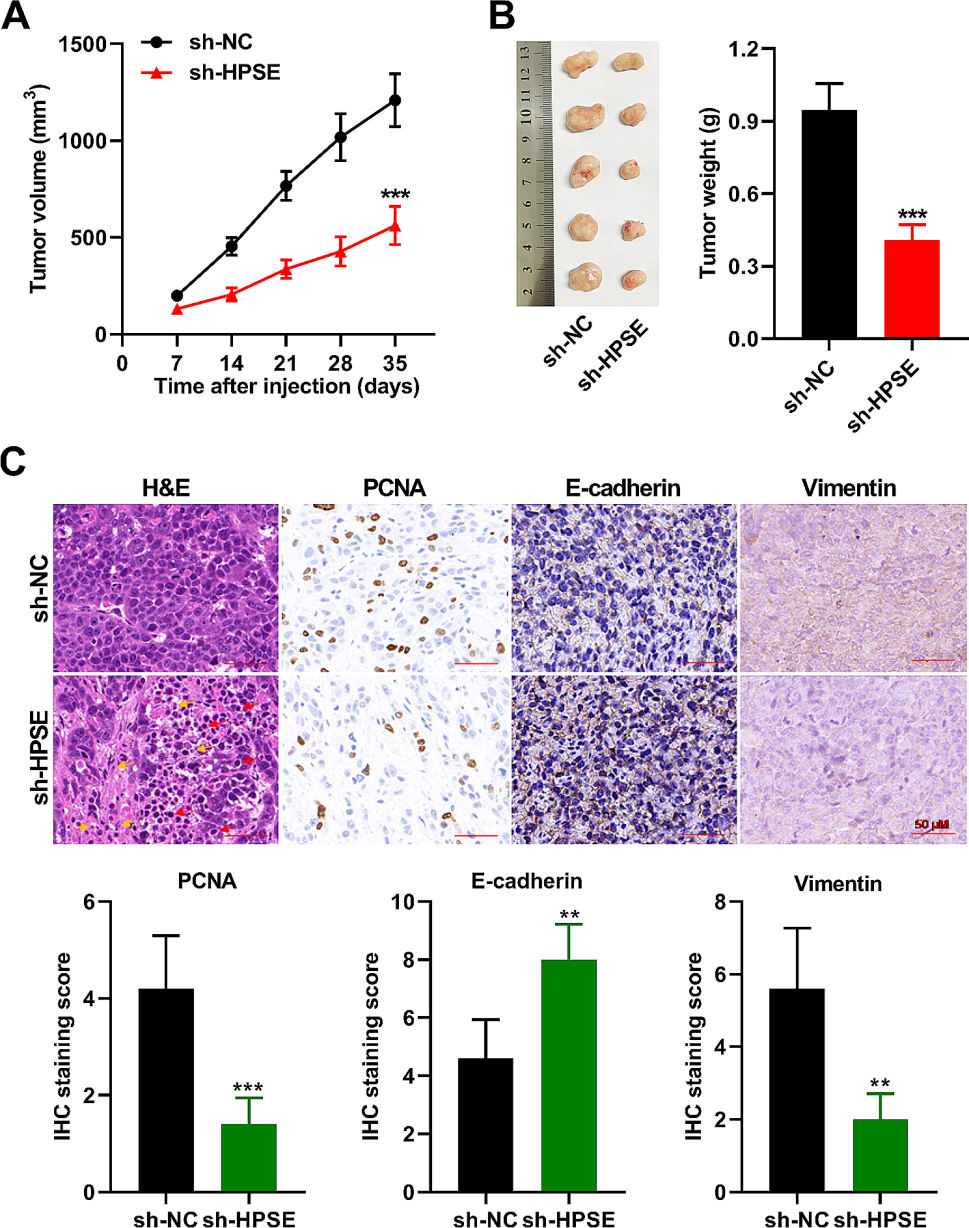
Knockdown of HPSE inhibits tumor growth of glioma cells in vivo. (**A** and **B**) Changes in tumor volume and weight were shown. (**C**) The histological morphology of tumor tissue was detected by HE staining. Red arrows point to apoptotic cells with nuclear shrinkage. Yellow arrows point to necrotic cells with granular and vacuolar degeneration. Relative protein levels of PCNA, E-cadherin, and vimentin, as quantified using IHC. *n* = 5. **P < 0.01, ***P < 0.001

**Fig. 8:**
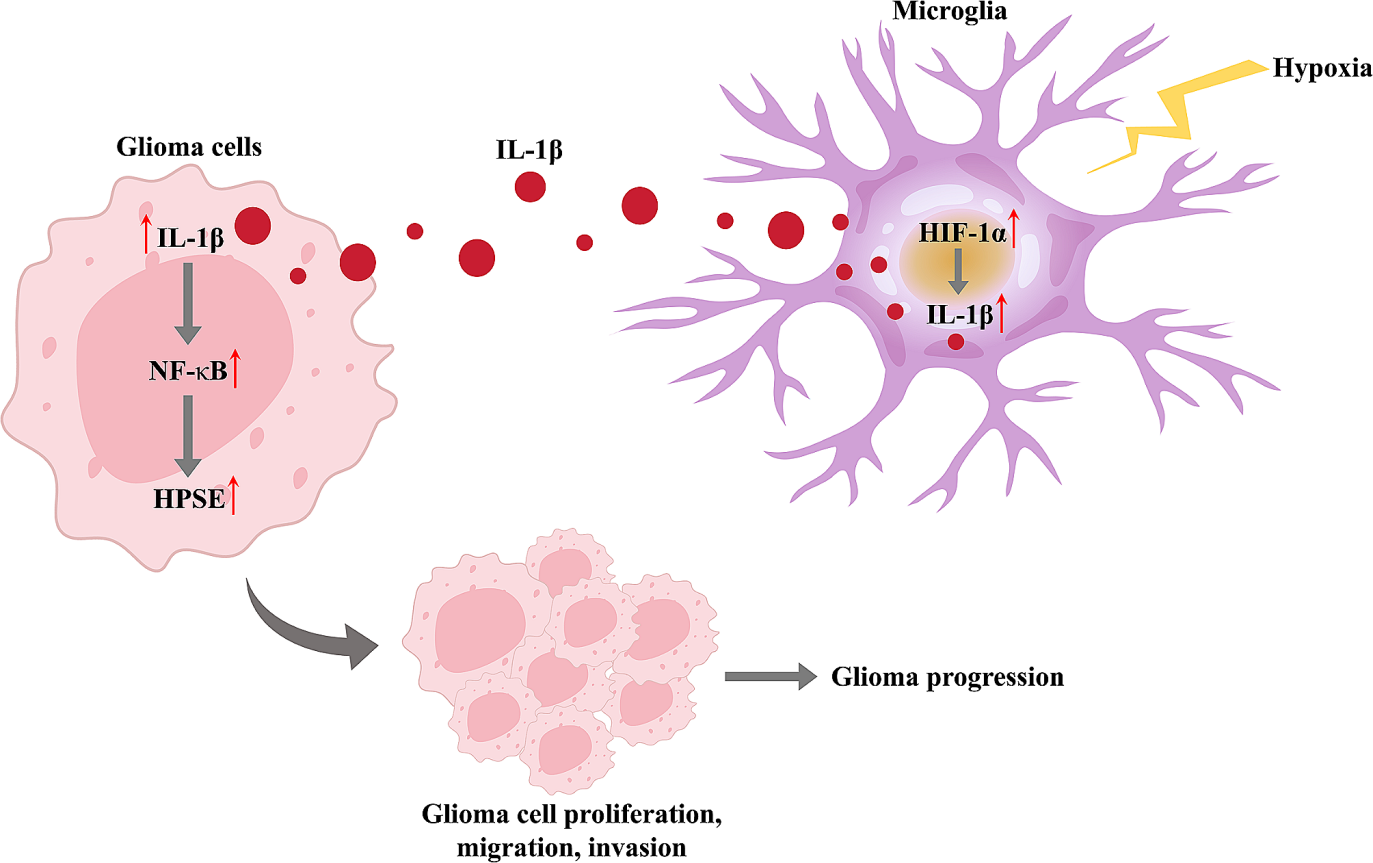
Schematic illustration of the molecular mechanism by which the hypoxic microenvironment promotes glioma progression. Hypoxic microglia-secreted IL-1β promotes glioma cell proliferation, migration, and invasion through NF-κB-mediated upregulation of HPSE expression

To verify the carcinogenic role of HPSE in glioma, human glioma xenograft in nude mice was applied. As displayed in Fig. 7A and B, glioma growth was significantly slowed and tumor weight was reduced when HPSE expression was knockdown. Moreover, abnormal histological pattern, including disordered arrangement, nuclear shrinkage, and more apoptotic or necrotic cells, was appeared in the sh-HPSE group. The activities of PCNA and vimentin protein were subdued, but E-cadherin was increased in the tumor cells with HPSE downregulation relative to the control tumor tissues (Fig. 7C). The results of these animal experiments unfolded that silencing HPSE curbed the proliferation and metastasis of glioma in mice. Taken together, our in vitro and in vivo findings suggested that hypoxia-induced activation of HIF-1α/IL-1β axis in microglia promotes glioma progression via NF-κB-mediated upregulation of HPSE expression (Fig. 8).

## Discussion

In this research, we built cell and mouse models and found that hypoxic HMC3 promoted glioma cell proliferation, migration, and invasion. At the mechanistic level, we discovered that activation of HIF-1α stimulated IL-1β secretion by microglia under hypoxic condition and then IL-1β increases HPSE expression in glioma cells by activating NF-κB. Further findings suggested that hypoxia-induced activation of HIF-1α/IL-1β axis in HMC3 cells promoted glioma progression through NF-κB-mediated upregulation of HPSE expression. This is the first report on the cancer-promoting function and mechanism of hypoxic microglia-derived IL-1β in glioma. In addition, we identified a novel mechanism underlying the cancer-promoting activity of HPSE in the hypoxic microenvironment of glioma.

Hypoxia is a central factor affecting the proliferation and metastasis of gliomas. Gliomas are malignant tumors typically located in the brain, formed from glial cells that are highly dependent on oxygen [22]. It has been reported that acrolein produced by glioma cells under hypoxic conditions suppresses anti-tumoral activities by inhibiting neutrophil AKT activity and promoting anti-inflammatory phenotype [23]. Under hypoxic conditions, glioma cells activate the phosphatidylinositol-3-kinase/AKT/HIF-1α signaling pathway and adaptive mechanisms to survive and proliferate, promoting tumor growth [15]. Li et al. [24]. and Jensen et al. [25]. confirmed that hypoxia can stimulate the production of VEGF, promote invasion and metastasis of glioma cells, increasing the difficulty of treatment. Therefore, understanding the effects and regulatory mechanism of hypoxia on the tumor microenvironment is important for the diagnosis and treatment of glioma. Currently, new treatment strategies are being developed that target hypoxia in the tumor microenvironment, including targeting tumor angiogenesis, radiotherapy, hypoxic radiotherapy, and immunotherapy [26, 27]. Among them, drugs targeting VEGF have been extensively researched and utilized [28]. VEGF is an important factor that induces tumor angiogenesis and maintenance under hypoxic conditions. Anti-VEGF drugs such as bevacizumab and ziv-aflibercept can inhibit the VEGF signaling pathway, thereby suppressing tumor angiogenesis and growth and reducing the impact of hypoxia on glioblastoma cells [29, 30]. Additionally, Chiara et al. [31]. reported that the treatment of glioblastoma cells with hyperbaric oxygen could inhibit cell proliferation and reduce the expression of HIF-1α. However, research on hypoxic-related targeted drugs for glioma is far from sufficient. Therefore, we conducted an in-depth study of the molecular mechanisms of hypoxia regulating microglia cells and ultimately regulating glioma progression.

Under hypoxia conditions, microglia cells may lose their immunomodulatory function, leading to tumor immune escape and suppression of anti-tumor immune response [32]. Besides, hypoxia may promote the alteration of microglia from M1 type to M2 type, which leads to the transformation of microglia from antitumor active cells to tumor supportive cells, thus increasing tumor growth and spread [20, 32]. Rackele et al. [33]. revealed that microglial LPA could promote glioblastoma growth and migration. Keitaro et al. [34]. demonstrated that IL-1β from microglia enhanced glioblastoma growth by modulating the STAT3/NF-κB pathway. These evidences suggested that controlling the activity of microglia in hypoxic tumor microenvironment might be an important strategy in glioma therapy. In this research, we applied a microglia cell line HMC3 to mimic the effect of hypoxic microenvironment on malignant phenotype of glioma cells. We found that hypoxic HMC3 promoted the malignant biological phenotypes of U251 and U87 cells, which was comparable with the conclusions of Rackele et al. [33]. and Keitaro et al. [34]. . Our findings indicated that hypoxia management heightened IL-1β secretion by HMC3 cells by activating HIF-1α. IL-1β from hypoxic HMC3 cells promoted proliferation, migration, and invasion of U251 and U87 cells. Herein, we accumulated further evidence of the tumor-promoting action of hypoxic microglia in glioma.

Our previous research has revealed that HPSE confers chemoresistance to temozolomide in glioma by promoting exosome-mediated the transfer of hsa_circ_0042003 from temozolomide-resistant glioma cells to sensitive cells [35]. Wu et al. [36]. reported that under the hypoxic condition, increases in HPSE mRNA, protein, and enzymatic activity were observed in human pancreatic cancer cells. Several papers have reported that NF-κB could upregulate transcription and content of HPSE by targeting its promoter and activating its transcriptional activity [37–40]. Therefore, we further explored the carcinogenic influence of HPSE and its underlying mechanisms in glioma within the hypoxic tumor microenvironment. We used BAY11-7085, an inhibitor of NF-kB signaling pathway, to test whether IL-1β-induced HPSE upregulation is also dependent on NF-κB activity in in vitro glioma cells. IL-1β-induced upregulation of HPSE expression in U251 and U87 cells was demonstrated to be attributed to the activation of NF-κB. Hypoxic HMC3 cells-secreted IL-1β facilitated the proliferation, migration, and invasion abilities of U251 and U87 cells via NF-κB-mediated upregulation of HPSE expression. Our findings further demonstrated that HIF-1α was overexpressed in HMC3 cells and thus upregulated IL-1β levels in HMC3 under the hypoxic condition. Following this, HMC3 cells could enhance the expression of NF-κB by secreting IL-1β into glioma cells, and the increased expression of NF-κB could up-regulate the abundance of HPSE and stimulate the malignant progression of glioma. The report on this novel mechanism underlying the tumor-promoting action of HPSE in the hypoxic microenvironment of glioma is the first time. Nevertheless, there are two limitations in the present study. Although we identified the functions of HPSE and its regulatory mechanisms within the hypoxic glioma microenvironment, it remains unclear whether HPSE is a good candidate as a diagnostic biomarker for patients with glioma. Furthermore, additional investigation is needed to verify the expression relationships of HIF-1α, IL-1β, NF-κB, and HPSE genes in clinical tumor samples. In the future, we will perform a multicenter study to validate the clinical significance of HPSE in glioma.

In summary, we established that hypoxia-induced activation of HIF-1α/IL-1β axis in microglia promoted glioma progression via NF-κB-mediated upregulation of HPSE. The findings of this study expand the theoretical basis of the pathogenesis of glioma and deliver important thoughts for the subsequent exploration and development of new drugs.

## Data Availability

No datasets were generated or analysed during the current study.

## Abbreviations

HIF-1: Alpha Hypoxia inducible factor-1α
VEGF: Vascular endothelial growth factor
FBS: Fetal bovine serum
DMEM: Dulbecco’s Modified Eagle’s Medium
CCK8: Cell Counting Kit-8
EdU: 5-ethynyl-2’-deoxyuridine
DAPI: 4’-6-diamidino-2-phenylindole
ELISA: Enzyme-linked immunosorbent assay
TNF-alpha: Tumor necrosis factor-α
MCAF: Monocyte chemotactic and activating factor
IL-6: Interleukin-6
IL-1α: Interleukin-1α
INF: Gamma interferon-γ
IL-1: Beta Interleukin-1β
IL-8: Interleukin-8
GM-CSF: Granulocyte macrophage colony stimulating factor
siRNA: Small interfering RNA
Lv: Lentiviral vector
shRNA: Short hairpin RNA
HPSE: Heparanase
NF-κB: Nuclear factor-κB
rhIL-1beta: Recombinant human interleukin-1β
HE: Hematoxylin-eosin
IHC: Immunohistochemistry
PCNA: Proliferating cell nuclear antigen
